# Acute Myocardial Infarction of the Left Main Coronary Artery Presenting with Cardiogenic Shock and Pulmonary Edema during Noncardiac Surgery

**DOI:** 10.1155/2021/5460816

**Published:** 2021-08-13

**Authors:** Sakae Takenaka, Takao Konishi, Tomoya Sato, Atsushi Tada, Takuya Koizumi, Yoshifumi Mizuguchi, Takahide Kadosaka, Ko Motoi, Yuta Kobayashi, Hirokazu Komoriyama, Yoshiya Kato, Miwa Sarashina, Kazunori Omote, Shingo Tsujinaga, Takuma Sato, Rui Kamada, Kiwamu Kamiya, Hiroyuki Iwano, Toshiyuki Nagai, Tatsuya Orimo, Hirofumi Kamachi, Akinobu Taketomi, Toshihisa Anzai

**Affiliations:** ^1^Department of Cardiovascular Medicine, Faculty of Medicine and Graduate School of Medicine, Hokkaido University, Sapporo, Japan; ^2^Department of Gastroenterological Surgery I, Faculty of Medicine and Graduate School of Medicine, Hokkaido University, Sapporo, Japan

## Abstract

Acute myocardial infarction (AMI) caused by severe stenosis of left main coronary artery (LMCA) presenting with cardiogenic shock and pulmonary edema during noncardiac surgery is uncommon, but a catastrophic event. A 77-year-old male with cholangiocarcinoma underwent hepatectomy. During the surgery, he presented with cardiogenic shock, which did not respond to infusion administration or vasopressor. A transesophageal echocardiogram revealed anterior, septal, and lateral severe hypokinesia and impaired left ventricular function. Emergent coronary angiogram showed severe stenosis of LMCA. The patient underwent primary percutaneous coronary intervention (PCI) under the support of intra-aortic balloon pump, followed by extracorporeal membrane oxygenation. The chest roentgenogram showed pulmonary edema. Two days after PCI, he successfully underwent hepatectomy and bile duct resection. Early identification of the cause of hemodynamic instability during noncardiac surgery and invasive strategy are important for minimizing the myocardial injury and improving clinical outcomes in AMI of LMCA.

## 1. Background

Acute myocardial infarction (AMI) of the left main coronary artery (LMCA) is a rare, but critical event [1]. Furthermore, AMI during noncardiac surgery is extremely rare [2]. As the LMCA provides three-fourth of the myocardial blood supply [3], acute stenosis or occlusion of the LMCA without sufficient collateral arteries is often complicated by cardiogenic shock or pulmonary edema. Early diagnosis and reperfusion therapy are associated with improved clinical outcomes in patients with AMI complicated with shock [2]. We report a case of AMI of the LMCA presenting with cardiogenic shock and pulmonary edema during noncardiac surgery.

## 2. Case Presentation

**Figure 1:**
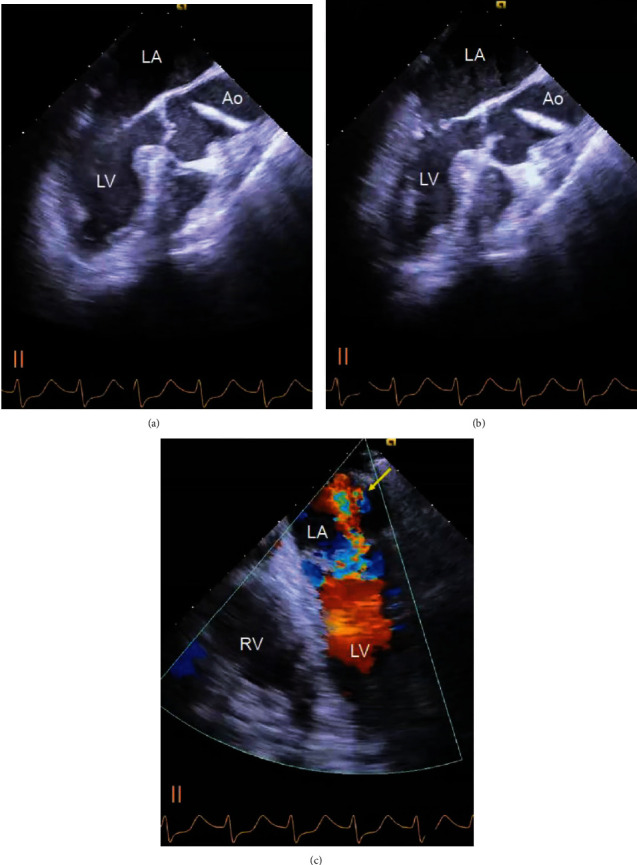
Transesophageal echocardiography. (a, b) Severe hypokinesis of the anteroseptal wall was observed at a 130° angle in a (diastole) and b (systole). Left ventricular ejection fraction was measured as 20%. LA: left atrium; LV: left ventricle; Ao: aorta; RV: right ventricle. (c) Severe mitral regurgitation at a 0° angle (arrow).

**Figure 2:**
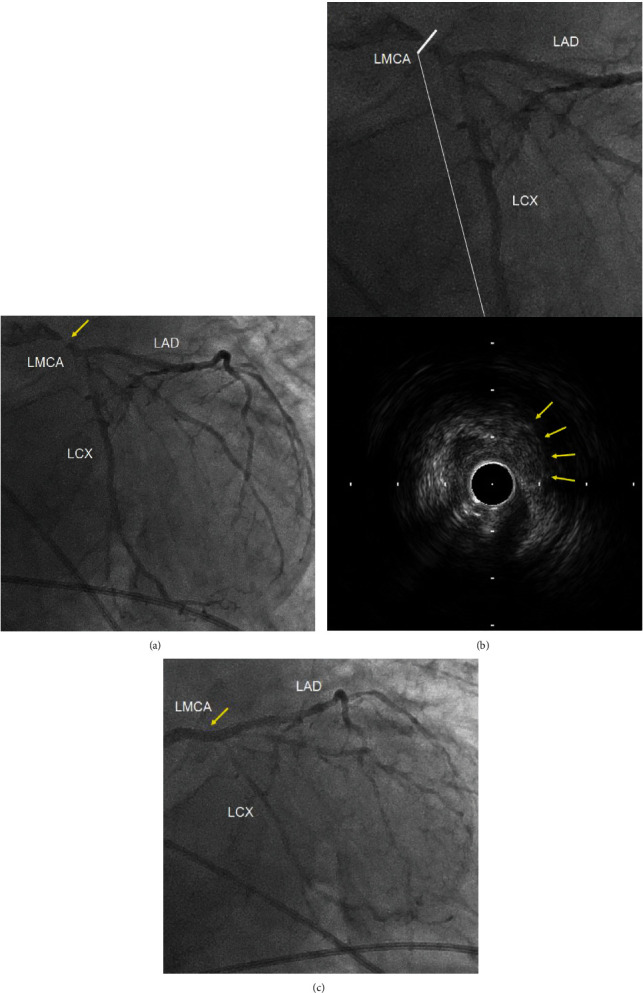
(a) Emergent coronary angiography. Right anterior oblique caudal view showing a severe stenosis of mid LMCA (arrow). LMCA: left main coronary artery; LAD: left anterior descending artery; LCX: left circumflex artery. (b) Intravascular ultrasound study. The section at the white line showing low attenuation plaque around the entire circumference of the vessel with ulceration (arrows) in the mid LMCA. LMCA: left main coronary artery; LAD: left anterior descending artery; LCX: left circumflex artery. (c) Final coronary angiography. Right anterior oblique caudal view showing no significant LMCA stenosis with grade 3 TIMI flow after stent implantation (arrow). LMCA: left main coronary artery; LAD: left anterior descending artery; LCX: left circumflex artery.

**Figure 3:**
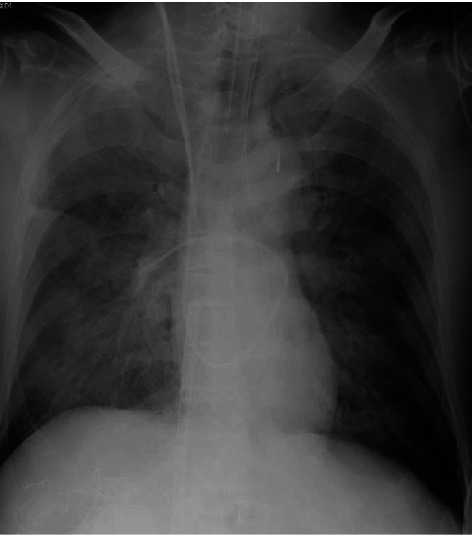
Chest roentgenogram. Chest X-ray showed a severe pulmonary edema.

A 77-year-old man with a history of hypertension and smoking underwent hepatectomy for cholangiocarcinoma. He had no angina symptoms prior to the surgery. In preoperative examination, a twelve-lead electrocardiogram (ECG) was normal, and no wall motion abnormality was detected on echocardiography. During the hepatectomy, his systolic blood pressure dropped to 40 mmHg and was not improved by rapid infusion administration and a vasopressor. He was referred to our department for identification of the cause of his refractory hypotension. On physical examination, he was intubated with body temperature 36.0° C, systemic blood pressure 40/- mmHg, and heart rate 120 bpm. His percutaneous oxygen saturation on artificial respirator (FiO_2_ 100%) was 90%. Laboratory tests revealed a 7.2 × 10^3^/mm^3^ white blood cell count, 10.4 × 10^4^/mm^3^ platelet count, 10.1 g/dL hemoglobin concentration, 170 IU/L creatine kinase, 22 IU/L MB fraction, 273 ng/L troponin-I, 3.09 *μ*g/mL d-dimer, 1.2 mg/dL total bilirubin, 1757 IU/L AST, 2647 IU/L ALT, 5158 IU/L LDH, and 0.06 mg/dL C-reactive protein serum concentration. The ECG showed ST depression in lead II, and a transesophageal echocardiogram (TEE) revealed severe hypokinesia of the anteroseptal and lateral wall, left ventricular ejection fraction (LVEF) of 20%, and severe mitral regurgitation (Figures 1(a)–1(c); see video 1 and 2 in Supplementary Material available online at https://drive.google.com/file/d/1RdC-7s-jwodX6bldxCBS1Kba2cS1DIjS/view and https://drive.google.com/file/d/1KsyF4WTUgB0X9wdrhtMetjzbgzYSdaSC/view, respectively). After abdominal closure, he was transferred to a near hybrid operation room. Coronary angiography (CAG) revealed severe stenosis of the left main coronary artery (LMCA) with a thrombolysis in myocardial infarction (TIMI) flow of grade II and moderate stenosis of the proximal segment of the left circumflex artery and ramus intermediate branch (Figure 2(a); see video 3 in Supplementary Material available online at https://drive.google.com/file/d/1LO67IkfRkyN1SEi9XlvNDTMhEjtHeG75/view). After the insertion of an intra-aortic balloon pump (IABP), emergent percutaneous coronary intervention (PCI) was performed. An intravascular ultrasound study (IVUS) of the LMCA revealed the presence of an attenuation plaque around the entire circumference of the mid portion of the LMCA (Figure 2(b); see video 4 in Supplementary Material available online at https://drive.google.com/file/d/1Iwo7m03maLjClM5FUgyT8rMfzQH3xj8o/view). The IVUS also showed ulceration and positive remodeling, suggesting an unstable plaque. The minimum lumen area of the LMCA lesion was 1.50 mm^2^. After IVUS, a predilatation was performed using a 2.5 × 15 mm semicompliant balloon. A drug-eluting stent (XIENCE Sierra® 3.0 × 33 mm, Abbott Vascular, Santa Clara, CA, USA) was implanted between the LMCA and the proximal left anterior descending artery. The final CAG showed optimal dilatation of the LMCA stent and a TIMI flow of grade III (Figure 2(c)). Extracorporeal membrane oxygenation (ECMO) was initiated to improve hypooxygenation due to severe pulmonary edema demonstrated by a chest roentgenogram (Figure 3). After the initiation of ECMO, the right heart catheterization showed that right atrial pressure, mean pulmonary arterial pressure, and pulmonary capillary wedge pressure were 9 mmHg, 24 mmHg, and 14 mmHg, respectively, suggesting the hemodynamic improvement. On the next day, ECMO was extracted due to the improvement of pulmonary edema. As the bile duct resection was not completed during the initial surgery, he had a high risk of intra-abdominal infection, which meant that it might be preferable to resume the hepatic surgery as early as possible. Two days after PCI, he successfully underwent hepatectomy and bile duct resection under the support of an IABP. The peak CK and CK-MB were 4296 IU/L and 438 IU/L, respectively. One month after PCI, LVEF was improved to 64% and mitral regurgitation was ameliorated from severe to mild. He remained free from chest pain or coronary ischemic events during hospitalization on a regimen of aspirin 100 mg/day and clopidogrel 75 mg/day until he died of sepsis three months after the surgery.

## 3. Discussion

This case illustrates the rare characteristics of AMI of the LMCA complicated by cardiogenic shock and pulmonary edema during a noncardiac surgery, which were successfully treated by PCI under the support of IABP and ECMO. AMI in the LMCA is a rare clinical event, with incidences of 0.9% to 5.2% according to previous studies of patients with AMI undergoing CAG [1, 4]. Among AMI patients with cardiogenic shock undergoing PCI, the prevalence of the LMCA as the culprit vessel is 1.3% to 9.5% [5–7]. Smilowitz et al. reported that perioperative AMI was observed in 84,093 (0.88%) of 9,566,277 patients who underwent noncardiac surgery and more than half of them were associated with vascular or orthopedic surgery [2]. Therefore, intraoperative AMI of the LMCA, complicated by cardiogenic shock and pulmonary edema, during hepatectomy for cholangiocarcinoma is extremely rare.

The etiology of AMI in this case might be due to a thrombotic cause, classified as a type 1 MI, while the dominant mechanism of perioperative acute coronary syndrome is demand ischemia, which is classified as a type 2 MI [8]. Helwani et al. showed that most perioperative AMIs were adjudicated as type 2 MI (73%) compared to type 1 MI (25%) from CAG findings [8]. In this case, the IVUS showed a stenotic lesion with ulceration in the midportion of the LMCA (Figure 2(b)). Persistent pain due to surgical invasion might have caused increased sympathetic nervous system activity, leading to increased cardiac afterload. Bleeding and hypovolemia during surgery could have caused anemia and tachycardia. In addition to these hemodynamic and physiological changes, acute inflammation and a prothrombotic state during surgery might have caused plaque rupture and acute thrombotic events in this case.

Early diagnosis and interventional strategies are needed to improve the prognosis of AMI of the LMCA presenting with cardiogenic shock and/or pulmonary edema. In addition to electrocardiographic monitoring, TEE might be useful, although not routinely used, for detecting myocardial ischemia by identifying regional wall motion abnormalities, even in a noncardiac surgery [9]. Because twelve-lead ECG is often not available due to operative positions or sterile fields, TEE could reveal abnormal wall motion in response to persistent intraoperative hemodynamic instability. Actually, in this case, twelve-lead ECG and transthoracic echocardiography could not be performed because the precordial lead placement and echocardiographic windows were in the proximity of the sterile fields. Smilowitz et al. showed that invasive management, such as PCI or coronary artery bypass grafting, was associated with lower in-hospital mortality than conservative management in a propensity-matched cohort of 34,650 patients with perioperative AMI (8.9% vs. 18.1%, *P* < 0.001) [2]. Furthermore, delays to reperfusion were associated with larger infarct size and 1-year mortality in patients with large anterior myocardial infarction [10]. Therefore, emergent primary PCI in a hybrid operation room, located near the original operation room, might be a reasonable treatment for patients with AMI of LMCA during noncardiac surgery.

A bleeding is one of the major concerns particularly in liver surgery because a lot of blood passes through the liver. Previous studies have shown that a hemorrhage and the need for a transfusion not only adversely affect perioperative outcomes but also the long-term prognosis in patients undergoing a hepatectomy for primary or secondary malignancies [11, 12]. If this case had undergone hepatectomy under the management of ECMO, adequate heparin would have been needed to prevent thrombotic complications within the circuit of ECMO. Due to the systemic anticoagulation, the volume of blood loss and volume of blood transfusions would have been increased during and after the operation. Although the vessel occlusion technique reduces blood loss during the operation, it is directly correlated with a high risk of postoperative hepatic failure [13]. In this case, the LVEF was improved from 20% to 40% two days after the emergent PCI and the roentgenogram also showed the improvement of pulmonary edema. These improvements of hemodynamic and respiratory conditions enabled the resumption of hepatectomy without ECMO. For these reasons, the hepatectomy and bile duct resection were performed two days after emergent PCI.

## 4. Conclusions

Early identification of the cause of hemodynamic instability during noncardiac surgery and emergent primary PCI might minimize myocardial injury and improve clinical outcomes in AMI of the LMCA.

## Supplementary Materials

Supplementary 1 Video 1: two-dimensional transesophageal echocardiography, three-chamber view showing a severe hypokinesia of the anteroseptal wall.

Supplementary 2 Video 2: two-dimensional transesophageal echocardiography, four-chamber view showing severe mitral regurgitation.

Supplementary 3 Video 3: emergent coronary angiography, left anterior oblique cranial view showing a severe stenosis of the mid portion of the left main coronary artery.

Supplementary 4 Video 4: intravascular ultrasound study showing low attenuation plaque with ulceration and positive remodeling in the mid portion of the left main coronary artery.
